# Children’s looking preference for biological motion may be related to an affinity for mathematical chaos

**DOI:** 10.3389/fpsyg.2015.00281

**Published:** 2015-03-17

**Authors:** Joshua L. Haworth, Anastasia Kyvelidou, Wayne Fisher, Nicholas Stergiou

**Affiliations:** ^1^Center for Autism and Related Disorders, Kennedy Krieger InstituteBaltimore, MD, USA; ^2^School of Health, Physical Education and Recreation, University of Nebraska OmahaOmaha, NE, USA; ^3^College of Public Health, University of Nebraska Medical CenterOmaha, NE, USA; ^4^Center for Autism Spectrum Disorders, University of Nebraska Medical CenterOmaha, NE, USA

**Keywords:** eye tracking, cross recurrence quantification analysis, sensorimotor, perception, complex systems

## Abstract

Recognition of biological motion is pervasive in early child development. Further, viewing the movement behavior of others is a primary component of a child’s acquisition of complex, robust movement repertoires, through imitation and real-time coordinated action. We theorize that inherent to biological movements are particular qualities of mathematical chaos and complexity. We further posit that this character affords the rich and complex inter-dynamics throughout early motor development. Specifically, we explored whether children’s preference for biological motion may be related to an affinity for mathematical chaos. Cross recurrence quantification analysis (cRQA) was used to investigate the coordination of gaze and posture with various temporal structures (periodic, chaotic, and aperiodic) of the motion of an oscillating visual stimulus. Children appear to competently perceive and respond to chaotic motion, both in rate (cRQA-percent determinism) and duration (cRQA-maxline) of coordination. We interpret this to indicate that children not only recognize chaotic motion structures, but also have a preference for coordination with them. Further, stratification of our sample (by age) uncovers the suggestion that this preference may become refined with age.

## Introduction

Developing children typically recognize biological motion in point-light displays (Fox and McDaniel, 1982). Interestingly, they specifically prefer to watch locomotion coherent with their own mode of locomotion; i.e., crawlers prefer to watch crawling whereas walkers prefer to watch walking (Sanefuji et al., 2008). Point-light studies such as this one provide insight into how children recognize and replicate movements of others in their social environment. The development of motor behavior relies, in part, on being able to incorporate the lessons learned from viewing others’ attempts at similar motor performance. By watching others, we are able to vastly multiply our own experience and knowledge of successful movement strategies. Several experiments have provided evidence that very young children are able to attribute intentionality and action goals to human models of motor behavior (Hofer et al., 2005; Klein et al., 2006; Hauf, 2007). Similar findings demonstrate the ability to distinguish intentional behaviors when viewing both familiar and novel motor actions (Woodward, 1998, 1999; Hofer et al., 2007; Jovanovic and Schwarzer, 2007; Jovanovic et al., 2007) and also when viewing both live and televised models of motor performances (Meltzoff, 1988a; Klein et al., 2006). In other studies, it has been shown that when the visual information is contrived to the point that it becomes unreliable, the reliance on this information for action-production is averted (Longo et al., 2008). These studies collectively point to the richness of a child’s perception of the movement behavior of other persons. Several additional investigations have sought to describe the nature and extent to which children are able to imitate movement behaviors of observed performers, either immediately or after a delay (Meltzoff, 1988b; Barr et al., 1996; Bremner, 2002; Herbert et al., 2006). Consistently, it is found that children ‘develop’ their ability to demonstrate delayed imitation throughout their early experiences, highlighting the complex milieu of sensory maturation, memory processes, and the formation of awareness of self and others.

Another key feature of these investigations that has received less attention is the notion that imitative behavior serves to directly foster the development of a rich motor behavioral repertoire, by eliciting motor repetition, and the production of potentially novel motor behaviors. From these findings others have built a case for the potential presence of a mirror neuron system in humans that is active from birth (Bertenthal and Longo, 2007). The differences in proposed mechanism behind the observed imitation are underpinned by the question of whether imitation is inherently an active or passive process. Regardless of the resolution of this mechanistic dispute, functional outcomes of motor imitation result in increases in motor behavioral experience.

We propose that imitation events are related to sensorimotor couplings resultant from the neural integration of oscillatory patterns of the viewed individual onto the viewer’s actions. Description of these temporal patterns involves a class of variables which are derived from mathematical chaos (Abarbanel, 1996), and are useful in describing the temporal structure of motion; including that of the individual point-lights provided in discrimination studies, posture, as well as human gait. Computations developed from chaos and dynamical systems theories (non-linear analyses) have demonstrated a unique capacity to characterize biological movement (i.e., posture, gait, and cardioballistics) according to its inherent temporal structure of motion variability (Glass and Mackey, 1988; Harbourne and Stergiou, 2003; Stergiou et al., 2004). Healthy biological motion exhibits a complex variability, meaning it is neither rigid nor random (Stergiou et al., 2006). The ability to perceive this complexity may be a discriminating factor in identifying biological motion from point-light displays.

Therefore, the current project focused on assessing the influence of perceived object motion on concurrent sensorimotor behavior of young children (age 4–6 years). The children were presented with an oscillating visual stimulus moving with various temporal structures; periodic, chaotic, and aperiodic. Gaze and posture responses to the stimulus motion were measured to determine whether information about the quality of its motion was able to guide the responsive strategies of these motor systems for gaze and postural movement control. We expect that children will demonstrate an ability to coordinate both gaze and posture to the various motion structures, with a particular affinity for more biologically relevant, chaotic motion. In order to pursue further resolution, we subdivided our cohort into age matched groups (4, 5, and 6 year-olds) to consider the possibility of developmental changes in sensorimotor responsiveness.

## Materials and Methods

### Participants and Procedures

**FIGURE 1:**
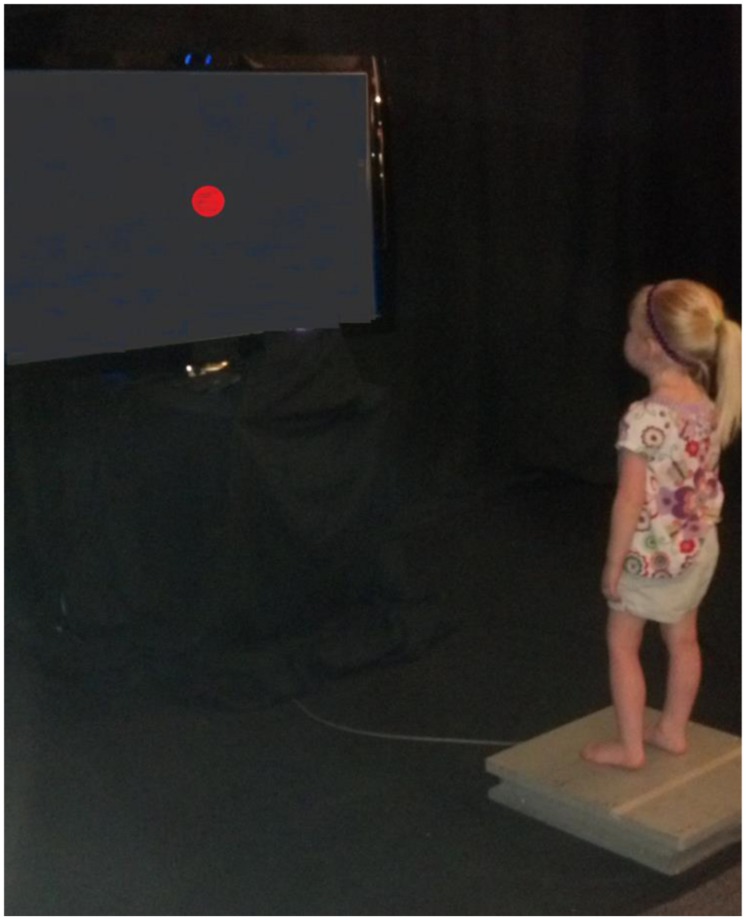
**Child participant during data collection, standing on a force platform while watching a point-light stimulus oscillate left–right on the display monitor (motion not shown)**.

Seventeen children participated in this study, ages 4–6 years. This included six boys and 11 girls, with average height of 112.8 ± 8.3 cm and average weight of 20 ± 5.8 kg. All children were verified to have normal vision and no neurological history. Typical development was confirmed using the Denver II scale (Frankenburg et al., 1992). Participants attended a single data collection session during which synchronous measures of eye movement and standing posture were collected while viewing a series of point-light stimulus motions (**Figure 1**). Children stood atop a force platform (Advanced Mechanical Technology Inc., OR6–7, with MSA-6 amplifier) where center of pressure data was collected at 50 Hz. Stimuli were presented on a 55′′ 1920 × 1200 pixel LCD display, with a black curtain surround to block sight of objects in the peripheral visual field. FaceLab 4.5 (Seeing Machines, Acton, MA, USA) eye-tracking equipment was mounted on the monitor stand, and was used to track eye movements also at 50 Hz. This sampling rate was selected as the highest common frequency across all equipment, and is also sufficient to capture the dynamics of the measured gaze and posture behaviors. Stimulus velocity was designed to prevent saccade or rapid postural perturbation. Custom LabView (National Instruments, Austin, TX, USA) software was designed to synchronize all data collection and stimulus display. Procedures were approved by the Institutional Review Board of the University of Nebraska Medical Center, and consent was obtained from the parent(s) of each child before participating.

### Stimulus Presentation

The motion of the stimulus differed across three conditions by scaling temporal complexity; including periodic (Sine), pseudo-periodic (Chaos), and aperiodic (Brown Noise) motion structures. Each signal was created in Matlab (MathWorks, Natick, MA, USA) with length of 10500 data points, which represents three and a half minutes of continuous stimulus motion when displayed at 50 Hz. The generated data series were displayed via the main Labview application during each testing condition. The Sine signal was generated with the embedded sin() function. This signal represents simple periodic redundancy, similar to what would be seen from a frictionless clock pendulum. Chaos is a complex signal that was created from the horizontal aspect of the distal position of a two-linkage, double-pendulum model which has previously been shown to emulate the dynamics of human posture (Suzuki et al., 2012). Shinbrot et al. (1992) assert that such a model would have sufficient degrees of freedom to afford chaotic dynamics. Surrogation analysis (Theiler et al., 1992) confirmed that the generated signal exhibited chaotic dynamics. Brown Noise demonstrates aperiodic dynamics, and is equivalent to integrated white noise (which is perfectly stochastic). Brown Noise was created by iterative perturbation of the position of the stimulus, by random direction and distance (within a specified boundary distance). This signal structure was selected as it provides an aperiodic motion structure, but also affords continuous smooth pursuit eye movement responses. Furthermore, early work by Collins and Deluca (1994) provided the observation that human posture expresses Brown motion.

### Data Processing

Trials lasted for three and a half minutes; with data recorded at 50 Hz. Gaze represents the on-screen pixel coordinate to which the participant was looking throughout the trial. Center of pressure was recorded as the measure of posture. Only mediolateral aspects of posture and gaze were further processed, to provide assessment of response to the horizontal motion of the stimulus. Gaze data was treated to remove blink events that occurred during collections. When the eye-tracker is unable to track the eyes, it reports a zero (0) value for gaze position. These values were removed from the time series, and replaced using a fifth order cubic spline (Matlab, interp1 function). Gaze and posture data were then filtered using a double pass Butterworth filter with a 10 Hz cutoff (Mathworks Inc.), which corresponds to previous observations of standing posture in children. Further processing included normalization and the identification of segments during which the child was not speaking or making overt motions with their head or arms. The longest common segment was 1500 data points, or 30 s of continuous data. These segments were selected and submitted for analysis. Cross recurrence quantification analysis (cRQA) was used to assess coupling of gaze (Gaze) and posture (COP) to the stimulus, separately, as well as between gaze and posture to gage sensorimotor coupling (SensMot).

### Cross Recurrence Quantification Analysis

The cRQA tests the relative likelihood of recurrence of behaviors across two time series in a common embedded phase space (Zbilut et al., 1998; Shockley et al., 2002; Shockley, 2005). Parameters of time delay (from average mutual information algorithm; Fraser and Swinney, 1986) and embedding dimension (from False Nearest Neighbors algorithm; Abarbanel, 1996) are used to unfold the signals into phase space (Takens, 1981). These algorithms indicated that values of 15 and 6, respectively, were appropriate for the data in this study. It must also be decided what proximity of two points would be required in order for them to be considered recurrent. This typically involves establishing a distance threshold, or radius (Shockley, 2005). We chose to algorithmically select the radius for each data series, such that a common rate of recurrence (5%) could be established across all data. This was opted to maintain consistency amongst all comparisons and to prevent saturation of determinism (Shockley, 2005). We evaluated the selected radius, group-wise and condition-wise using a 3 × 3 (Age × Stimulus) Mixed ANOVA; analogous in a way to using fixed radius and evaluating percent recurrence (**Table 1**). We also applied a minimum line length (minline) threshold of 25, such that only pairs of points which are contiguously recurrent for 25 time points will be considered to form lines of recurrence. At a sampling rate of 50 Hz, then, recurrent lines mean that the two data series are coordinated for a minimum of 500 ms, which will prevent short coincidental events, e.g., saccades, from being considered as coordination events. We also ran the COP and SensMot data with a more traditional minline value of 2, to allow better resolution of the postural data for percent determinism. Group- and condition- wise comparisons were similar regardless of the minline selected. Therefore, we present the results for minline of 25 for all signals, to maintain consistency.

**Table 1:** Condition-wise reporting of radius values selected to maintain 5% recurrence across all signal comparisons.

	Stimulus	Mean	SE
COP_ radius_	Sine	14.7	0.52
	Chaos	13.2	0.33
	Brown Noise	10.7	0.54
Gaze_ radius_	Sine	12.4	0.79
	Chaos	13.6	0.29
	Brown Noise	11.6	0.80
SensMot_ radius_	Sine	11.5	0.46
	Chaos	12.0	0.45
	Brown Noise	11.4	0.81

*COP_radius_ was found to differ across stimulus condition (*p* < 0.001, $\eta_{p}^{2}$ = 0.599, 100% observed power), with pairwise comparisons indicating differences between all conditions. No group-wise differences or interactions were found.*

The cRQA output includes percent determinism and maxline, representing probability, and duration (respectively) of recurrent behavior (Shockley et al., 2002; Shockley, 2005). Percent determinism is calculated as the ratio of recurrent points that form lines, divided by the total number of recurrent points; reported from 0 to 100%. So, if no lines are formed, then percent determinism will be 0%. Conversely if all recurrent points form lines, then percent determinism will be 100%. Maxline represents the longest bout of continuous recurrence between the two signals, measured in number of concurrent points. With minline set at 25, the smallest value of maxline possible is also 25. The upper limit is the length of the data in phase space, which would occur if the two behaviors were only coordinated throughout every time step of the trial. With data sampled at 50 Hz, each 50 point increment of maxline relates to 1 s of experiment time wherein the signals coordinate.

### Statistical Analysis

Separate 3 × 3 (Age × Stimulus) Mixed ANOVAs were used for statistical comparisons; for percent determinism and maxline for each of Gaze, COP, and SensMot. This design provided the opportunity to explore the primary hypothesis of the current study, regarding a main effect of stimulus. Planned, follow-up pairwise comparisons (LSD method) were used to identify where differences occurred, if any. The 2-way design additionally provided for assessment of the possible influence of age and/or interactions between age and stimulus. This second assessment is slightly more speculative and is underpowered for the current report, with only six 4 and 5 year-olds and five 6 year-olds, but provides strong support for extended investigations into the effect of age. Significance level was set to 0.05 for all comparisons.

## Results

### Stimulus

**FIGURE 2:**
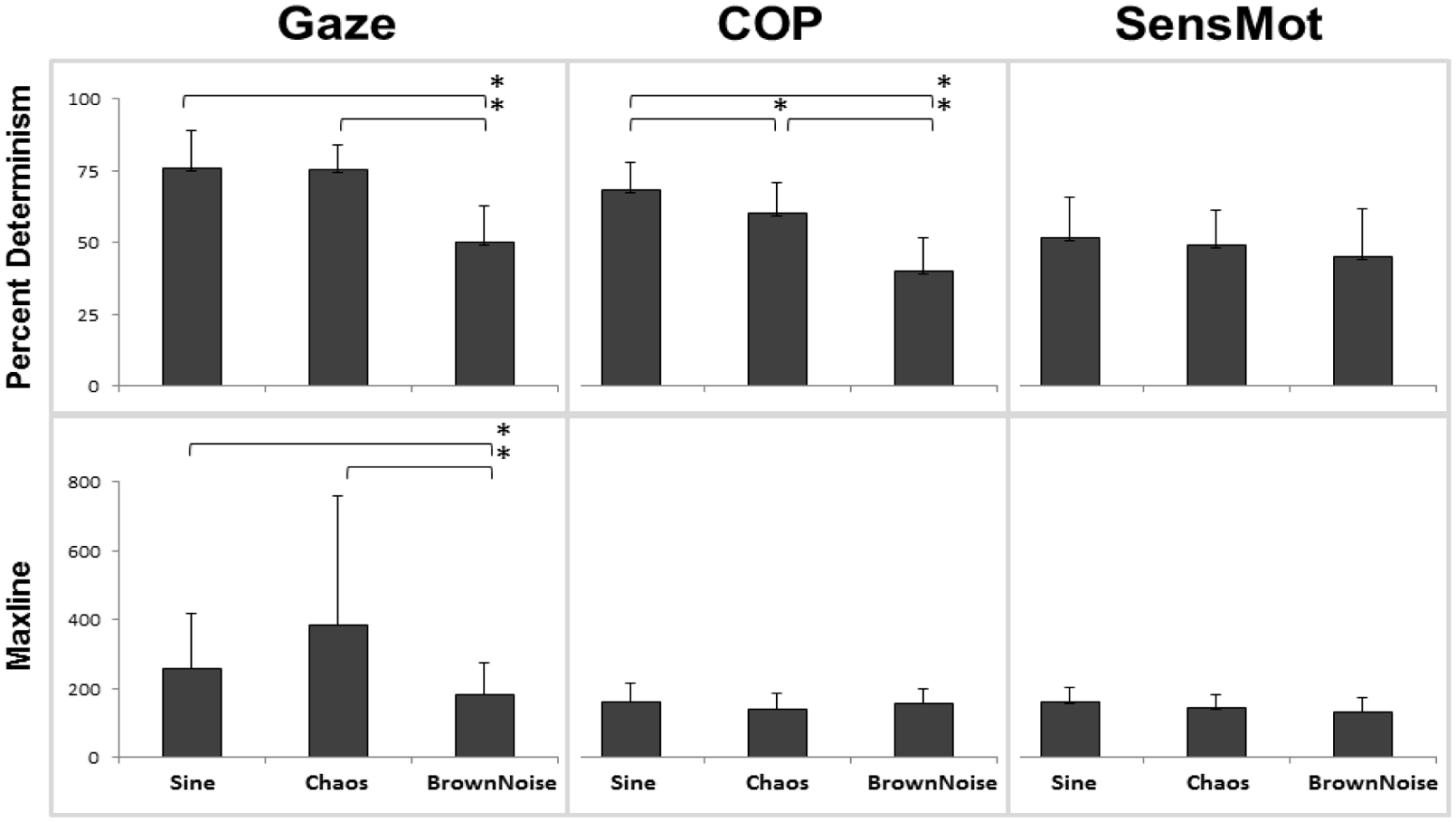
**Results from cross recurrence quantification analysis (cRQA); percent determinism is shown in the top row, with maxline across the bottom**. Each comparison is listed including Gaze (to stimulus), COP (to stimulus), and SensMot (Gaze to COP). From 3 × 3 ANOVA, only main effect of stimulus is shown here. *indicates differences with *p* < 0.05.

The statistical analysis produced significant results with respect to the main effect of stimulus (**Figure 2**). Gaze significantly responded to the structure of the stimulus motion with changes in percent determinism (*p* < 0.001, $\eta_{p}^{2}$ = 0.691, 100% observed power). Planned, follow-up testing indicated that rate of coordination (measured by percent determinism) of gaze to stimulus motion was similar in response to Sine and Chaos conditions (*p* = 0.906), but was lesser than each for the Brown Noise condition (*p* < 0.001 for both Sine and Chaos to Brown Noise). Duration of coordination (measured by maxline) also responded to the structure of stimulus motion (*p* < 0.028, $\eta_{p}^{2}$ = 0.225, 67.5% observed power). Follow up testing showed that the difference between Sine and Chaos was not significant (*p* = 0.132), however, differences between Sine and Brown Noise (*p* = 0.021) and between Chaos and Brown Noise (*p* = 0.040) were significant.

A significant stimulus effect was found for COP percent determinism (*p* < 0.001, $\eta_{p}^{2}$ = 0.749, 100% observed power), but not for COP maxline. Planned, follow-up testing indicated significant differences among each of the three conditions, with the greatest rate of coordination in response to the Sine stimulus and the least rate of coordination in response to the Brown Noise stimulus (*p* < 0.01 for all comparisons). No main effect of stimulus was found for SensMot, for either percent determinism or maxline.

### Age and Interactions

**FIGURE 3:**
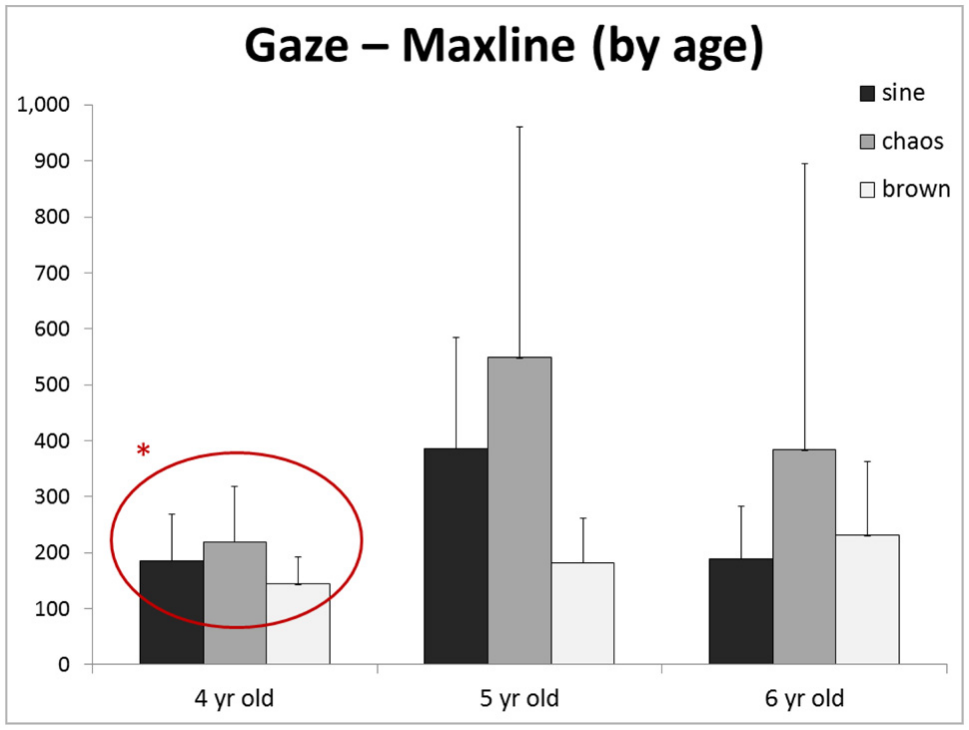
**Although no main effect of age (or interaction) was found, we noticed an interesting trend in the maxline of each age group**. Particularly, the 4 year-olds group appears to have the least response to the presence of chaotic dynamic*. Replication with larger group sizes could provide greater power, and thus potential identification of significant differences between age groups.

No main effect of age was found for any outcome for Gaze, COP, or SensMot. Moreover, no interactions were found for any outcomes for Gaze, COP, or SensMot. However, when examining closer the responses of each age to each stimulus (**Figure 3**), it is noticeable that the 4 year-old group seems to have consistently short maxline response to each stimulus and lacks an increased response to chaos. These observations were furthered explored as we looked into the main effect of age (*p* = 0.406, $\eta_{p}^{2}$ = 0.129, 28.3% observed power) and interaction of stimulus and age (*p* = 0.174, observed power = 34.2%), which showed neither observation to be significant.

## Discussion

Our results showed that typically developing children are able to coordinate aspects of their gaze and postural behaviors with the motion of a dynamic stimulus. Further, children appear to competently perceive and respond to chaotic motion, both in rate (percent determinism) and duration (maxline) of coordination. Being able to address complex motion might be a fundamental aspect of biological motion recognition. This elucidates a possible mechanism for the initial proposition that imitation behavior among children might be facilitated by an ability to perceive, and respond to, the chaotic complexity of viewed actions.

Chaotic motion is known to be inherent in human movement (Stergiou et al., 2006; Haworth et al., 2013), and may serve as a critical perceptive feature in biological motion recognition. It is important to know if only general features of motion are observed, though, or if even the minutia of movement variability is available to be considered by a viewer. Our results argue an ability to coordinate gaze, and in some respects posture, even with complex motion structures; echoing the complex stimulus behavior even at the level of fine detail. The lack of similar responses to brown noise indicate that motion structure coordination is possible as long as some fashion of deterministic order can be found in the stimulus motion. This may relate to the intentionality found in human movements, which operates through potentially infinite degrees of freedom to become realized as actual movements (Bernstein, 1996).

The critical benefit of chaotic dynamics may be its underlying deterministic variability; in other words, its non-random unpredictability. Riley and Turvey (2002) provide extensive support to the notion that movement variability represents exploratory strategies, not ‘noise,’ and is a characteristic feature of a healthy mover. Exploration is also well known to be an integral part of the child’s experience (Campos et al., 2000). It is important that children attempt new skills in new ways, finding their way to strategies that work best for themselves within their own self-typical environments (Adolph and Joh, 2009). Observation of others can be a huge informant to this process, allowing a child to access the action-effects of particular behaviors through a sort of egocentric proxy (Siegler, 1996; Siegler et al., 2010). Observation and imitation of others is both a natural and efficient way to gain added movement experience, conceivably even at the fine detail level of the particular movement. Bertenthal and Longo (2007) purport that a mirror neuron system operates in this direct mapping of viewed movements onto the motor cortex of the viewer; suggesting that even highly complex motions can be mapped in real-time.

Following this line of thought, we expected to find strong coupling of posture to viewed motion dynamics, particularly in response to chaotic motion. We found, though, that children did not seem to transfer the viewed motion structure to the regulation of posture in the way expected. Children generally coordinated posture with any stimulus for up to 200 data points at a time, which works out to roughly 4 s, and showed no difference across stimuli with regard to duration of coordination (maxline). Percent determinism showed, though, that posture was generally more often coordinated with the rhythmic stimulus and least coordinated with the arrhythmic stimulus. We contend that there are two possible explanations for this phenomenon. Firstly, the nature of the task may have not allowed for coupling of posture to the stimulus. We should note here that the instructions given to the subjects were to stand on the force plate as naturally as possible and look at the screen. No instruction was given to them on tracking their gaze or body with the stimulus, which may explain the lack of continuous coordination of posture with the visual stimulus. Secondly, the inertial effects may have influenced the ability to coordinate posture with the nuanced variance of the chaotic motion stimulus. In contrast to the eye, the body takes considerably more force and may utilize longer time delay reflexes to move. These results partially echo those of Scharli et al. (2012), who found weaker sensorimotor coordination in children of this age and evidence of a mechanical component. They argue that vision and posture should be regarded as individual systems, with *only mechanical interdependence* (Scharli et al., 2012). Our data show intermittent, non-trivial coordination (up to four continuous second bouts) of posture to stimulus, which provides evidence of active sensorimotor coordination and thus *dynamic informational interdependence*. Strong evidence for such sensorimotor coupling has been found in adults during both posture and gait (Kay and Warren, 2001; Stoffregen et al., 2006; O’Connor and Kuo, 2009; Giveans et al., 2011). It seems plausible, that the results of our current study imply an immature system that is yet unable to coordinate sensorimotor processes successfully over long durations. Hong et al. (2008) discuss at length the evolution of complexity and internal coordination of posture during sitting. They show that through aging (from children at 6 years, to children at 10 years, to young adults at 18–23 years) there seems to be a release of control over internal degrees of freedom, which leads to a relatively more adaptable posture. Possibly, children merely have a shorter sensorimotor coordination ‘attention’ span than adults.

This provokes the question of whether cognitive awareness (‘attention’) is necessary to translate viewed information into subsequent movement behavior. Olivier et al. (2008) reported that children ranging from 4 to 11 years of age show considerable changes in the control of posture, with a compounding influence of attention. Donker et al. (2007) have reported similar observations that attention affects postural organization. Our results of gaze maxline response to various motion structures (**Figure 3**) reveal the possibility that younger children possess a less diverse attention than their older peers. We wonder if, in line with the work of Hong et al. (2008), development brings with it a general complexification of behaviors; including posture and gaze. If so, this may confound our perspective of the results of the current study regarding sensorimotor coordination.

Finally, this work could become a point of focus for those looking to foster the development of a child. Therapists could potentially focus on the development of sensorimotor ‘attention’ as an indirect means toward increased functional movement behavior. Essentially, encourage children to take advantage of behavioral imitation to motivate the acquisition of motor experience in ways that increase awareness of successful variations of movement strategies.

## Conflict of Interest Statement

The authors declare that the research was conducted in the absence of any commercial or financial relationships that could be construed as a potential conflict of interest.
